# Effect of *trans*-Cinnamaldehyde on Methicillin-Resistant *Staphylococcus aureus* Biofilm Formation: Metabolic Activity Assessment and Analysis of the Biofilm-Associated Genes Expression

**DOI:** 10.3390/ijms21010102

**Published:** 2019-12-22

**Authors:** Barbara Kot, Hubert Sytykiewicz, Iwona Sprawka, Małgorzata Witeska

**Affiliations:** Institute of Biological Sciences, Faculty of Exact and Natural Sciences, Siedlce University of Natural Sciences and Humanities, 14 Bolesława Prusa Str., 08-110 Siedlce, Poland; hubert.sytykiewicz@uph.edu.pl (H.S.); iwona.sprawka@uph.edu.pl (I.S.); malgorzata.witeska@uph.edu.pl (M.W.)

**Keywords:** MRSA, biofilm, *trans*-cinnamaldehyde, metabolic activity, expression of genes, quantitative real-time PCR

## Abstract

The effects of *trans*-cinnamaldehyde (TC) on transcriptional profiles of biofilm-associated genes and the metabolic activity of two methicillin-resistant *Staphylococcus aureus* (MRSA) strains showing a different degree of adherence to polystyrene, were evaluated. Metabolic activity of *S. aureus* in biofilm was significantly decreased in the presence of TC at 1/2 minimum biofilm inhibition concentration (MBIC). Expression levels of the genes encoding laminin binding protein (*eno*), elastin binding protein (*ebps*) and fibrinogen binding protein (*fib*) in the presence of TC at 1/2 MBIC were lower than in untreated biofilm in both the weakly and strongly adhering strain. The highest decrease of expression level was observed in case of *fib* in the strongly adhering strain, in which the amount of *fib* transcript was 10-fold lower compared to biofilm without TC. In the presence of TC at 1/2 MBIC after 3, 6, 8 and 12 h, the expression level of *icaA* and *icaD*, that are involved in the biosynthesis of polysaccharide intercellular adhesin, was above half lower in the weakly adhering strain compared to biofilm without TC. In the strongly adhering strain the highest decrease in expression of these genes was observed after 3 and 6 h. This study showed that TC is a promising anti-biofilm agent for use in MRSA biofilm-related infections.

## 1. Introduction

Methicillin-resistant *Staphylococcus aureus* (MRSA) are responsible for hard-to-treat infections among different patient populations globally, and pose a growing problem for human health. MRSA are recognized as resistant to β-lactam antibiotics, but often MRSA strains also show resistance to many commonly used antibiotic groups, such as aminoglycosides, fluoroquinolones, macrolides, tetracycline and chloramphenicol [1]. The limited treatment options of MRSA infections result in higher mortality and increased financial costs. It was found that in the US, MRSA annually cause 80,000 invasive infections and between 11,000 and 18,000 deaths [2]. Biofilms are currently recognized as one of the most relevant factors of persistent infections, and make a major challenge for clinicians and clinical microbiologists. Biofilms are involved in chronic infections in all tissues of the human body. Multiple layers of bacteria in biofilm, enclosed in a self-produced exopolysaccharide glycocalyx, typically show no sensitivity to antibiotic therapy and host immune response.

Antimicrobial resistance of biofilms results from several mechanisms such as reduced antibiotic penetration, different growth rates of bacterial cells, nutrient gradients within the biofilm and the presence of dormant variants (persister phenomenon) highly tolerant to antibiotics. Other mechanisms of the antimicrobial resistance of biofilm are induced in the presence of antibiotics—they include different expressions of conventional and biofilm-specific antibiotic resistance genes and mutational mechanisms [3]. 

It was shown that bacterial cells in biofilm can tolerate up to 10–1000 times higher concentrations of antimicrobials than planktonic cells [4]. For this reason, there is a need to find alternative therapies to control infections caused by MRSA biofilm; e.g., compounds derived from plants. Treatment of infections with plant metabolites seem to be a reasonable alternative to antibiotics, and the return to the use of phytochemicals as antimicrobial agents is a result of pathogen resistance to any antibiotics that are used excessively or inappropriately. 

In our earlier research, we investigated the transcriptional profiles of specific staphylococcal genes (*eno*, *ebps*, *fib*) encoding microbial surface components recognizing adhesive matrix molecules (MSCRAMMs) and genes from the *ica* operon (*icaA* and *icaD*) in different time points during biofilm formation and under planktonic conditions. We showed that the expression of these genes was significantly higher in biofilm than in planktonic conditions [5]. The *eno* gene encodes α-enolase able to bind to laminin, and also acting as a plasminogen receptor. Binding of *S. aureus* to laminin, which is a major component of the basal membrane of the blood vessels, allows staphylococcal cells for adherence to their walls and the dissemination of bacterial cells by blood, initiating tissue colonization in different sites of the host [6]. The *ebps* gene encodes the elastin binding protein of *S. aureus* (EbpS). Elastin is a major component of the elastic fiber of the extracellular matrix (EM), and the ability of *S. aureus* to bind to this component promotes colonization of mammalian tissues [7]. The fibrinogen binding protein (Fib) encoded by the *fib* gene allows *S. aureus* to adhere to fibrinogen, which is present in the blood, and mediates platelet adherence, aggregation and clotting in sites of injury. Fibrinogen is one of the main proteins deposited on implanted biomaterials. Adhesion of *S. aureus* to fibrinogen leads to wound infection, colonization of implanted biomaterials and endocarditis [8]. Polysaccharide intercellular adhesin (PIA) encoded by *ica* operon is an adhesin responsible for the accumulation of bacterial cells in biofilm. PIA is the primary determinant promoting adhesive interactions between bacterial cells, and was demonstrated to be necessary for biofilm formation [9,10]. PIA is composed of β-1,6-linked-N-acetylglucosamine and fraction-containing non-N-acetylated-d-glucosaminyl [11,12]. Gene *icaA* encodes N-acetylglucosaminyl-transferase of low activity. Whereas, coexpression of the *icaA* gene and *icaD* significantly increases the activity of this enzyme and slime production [13]. Activity of these genes is essential for staphylococcal biofilm formation, therefore looking for alternatives to antibiotics that prevent biofilm formation, we investigated the effects of *trans*-cinnamaldehyde (TC) on the transcriptional profiles of these genes, and on metabolic activity during the biofilm formation of two MRSA strains showing different degrees of adherence to polystyrene. 

## 2. Results

### 2.1. Antibacterial Activity of TC against MRSA Strains

The antibacterial activity of TC against two MRSA strains showing different degrees of adherence to polystyrene was evaluated in vitro by the measurement of minimum inhibitory concentrations (MIC), minimum bactericidal concentrations (MBC), minimum biofilm inhibition concentrations (MBIC) and minimum biofilm eradication concentrations (MBEC) values. The susceptibility of the weak biofilm producer (strain 1037) to TC under planktonic conditions was higher compared to the strong biofilm producer (strain 27,887), and values of MIC and MBC for this strain were 0.06 mg/mL. In case of the planktonic cells of the strong biofilm producer, higher concentrations of TC were required to inhibit growth (0.24 mg/mL) and obtain a bactericidal effect (0.48 mg/mL) (Table 1). Biofilm growth of the weakly adhering strain was inhibited at 0.12 mg/mL (MBIC) of TC, and total eradication of the biofilm was observed at 1.92 mg/mL (MBEC). 

In case of the strongly adhering strain, concentrations of TC required to inhibit and eradicate biofilm cells were 0.48 and 0.96 mg/mL, respectively (Table 1).

### 2.2. The Influence of TC on Metabolic Activity of Biofilms in Different Time Intervals

The influence of TC on the metabolic activity of MRSA bacterial cells in biofilm was investigated in four time points using TC at concentrations 1/8 and 1/2 MBIC (Figure S1).

**Figure 1:**
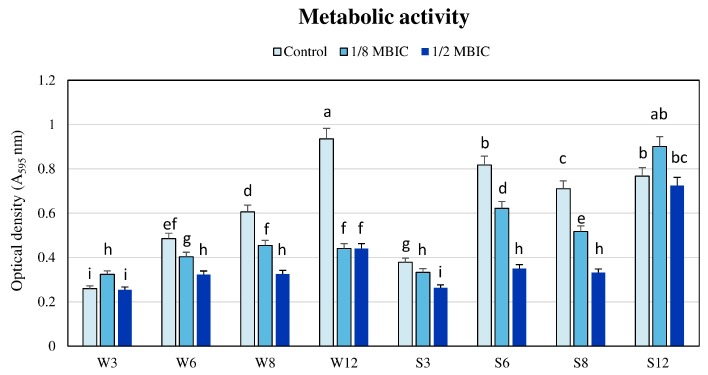
Metabolic activity of biofilms formed by MRSA strains in the presence of TC at concentrations 1/8 and 1/2 minimum biofilm inhibition concentration (MBIC) in different time intervals. Different letters (a, b, c, d, e, f, g, h, i, ab, bc, ef) denote significant differences in metabolic activity among investigated MRSA samples (Tukey’s test; *p* < 0.05). W: weak producer of biofilm; S: strong producer of biofilm; 3, 6, 8, 12: time of bacterial growth (h), MBIC: minimum biofilm inhibition concentration. Each assay was performed three times and the results were averaged.

Reduction of metabolic activity of the biofilm formed by a strongly adhering MRSA strain after 3, 6 and 8 h of TC treatment with 1/8 MBIC compared to the control values at the same time were 11.3% ± 4.8%, 23.9% ± 3.6% and 27.2% ± 4.2%, respectively, and metabolic activity of this strain in these time points was significantly lower than in biofilm that was non-exposed to TC (Figure 1). However, after 12 h, the biofilm metabolic activity in the presence of 1/8 TC MBIC was higher than in the biofilm without TC. but the difference was insignificant at *p* < 0.05 (Figure 1).

Significantly higher metabolic activity was also observed in case of the weakly adhering strain after 3 h of TC treatment with 1/8 MBIC compared to untreated biofilm (Figure 1). However, metabolic activity of biofilm formed by this strain after 6, 8, 12 h was reduced by 16.8% ± 4.2%, 25.3% ± 4.9% and 52.9% ± 4.9%, respectively, compared to the control.

Reduction of the metabolic activity of the strongly adhering strain in the presence of TC at 1/2 MBIC after 3 h was 30.4% ± 4.6%, after 6 h—57.1% ± 2.0% and after 8 h—53.3% ± 1.7%, compared to the control. After 12 h of TC treatment the degree of reduction was lower (3.3% ± 1.2%), and metabolic activity did not significantly differ from the activity of untreated biofilm (Figure 1). In the case of the MRSA strain that was a weak biofilm producer, a significant reduction of metabolic activity of the biofilm in the subsequent time points occurred and reached the maximum of 53.1% ± 4.8% after 12 h.

### 2.3. Expression Levels of Genes Associated with Biofilm Formation in the Presence of TC Quantified by Real-Time qRT-PCR

**Figure 2:**
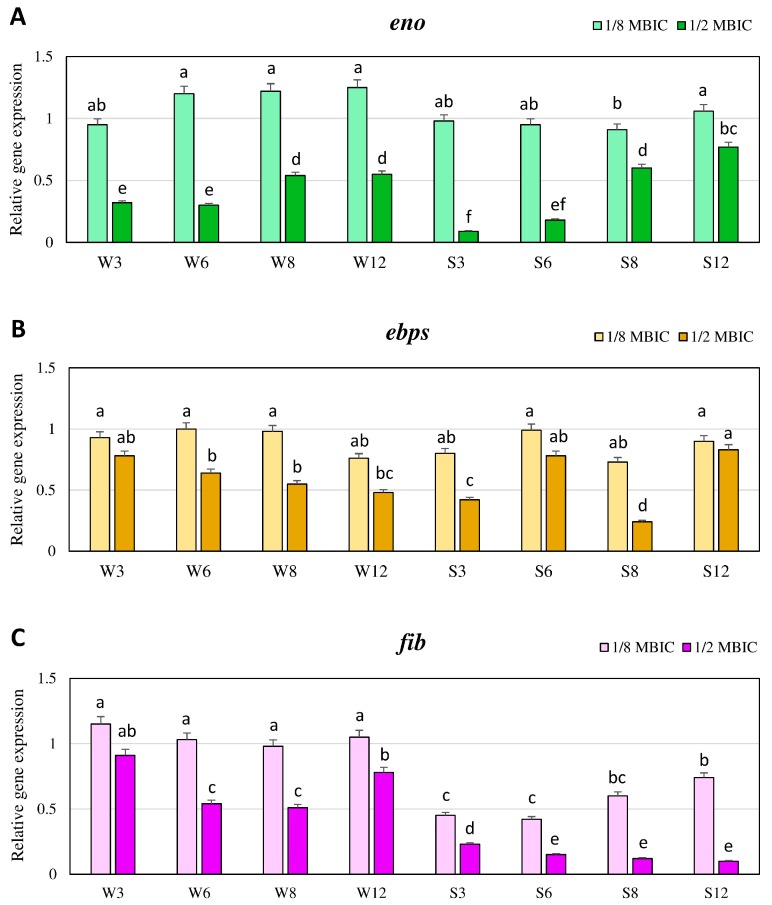
Transcriptional reprogramming of encoding laminin binding protein (*eno*) (**A**), elastin binding protein (*ebps*) (**B**) and fibrinogen binding protein (*fib*) (**C**) genes in MRSA strains treated with TC under biofilm growth condition. Gene expression data were normalized to the *rpoB* reference gene. Results are shown as *n*-fold changes (mean ± standard deviation (SD)) in the target gene expression compared to the control (biofilm untreated with TC). Significant differences in relative gene expression levels among examined MRSA samples were marked by different letters (a, b, c, d, e, f, ab, bc, ef) (Tukey’s test; *p* < 0.05). W: weak producer of biofilm; S: strong producer of biofilm; 3, 6, 8, 12: time of bacterial growth (h); MBIC: minimum biofilm inhibition concentration. Each assay was performed three times and the results were averaged.

Expression levels of three MSCRAMM genes (*eno*, *ebps* and *fib*) and two genes from *ica* operon (*icaA*, *icaD*) involved in biofilm formation were investigated. The expression patterns for these genes were followed after 3, 6, 8 and 12 h of growth, and they were compared between biofilm treated with TC at 1/8 and 1/2 MBIC concentrations, and biofilm that was nonexposed to TC. The results are presented as the *n*-fold change of gene expression levels in biofilm with TC in relation to the control. In the case of the weakly adhering strain, expression levels of *eno*, *ebps* and *fib* in the presence of TC at the concentration of 1/8 MBIC did not significantly differ in subsequent time points, and were similar as in the untreated biofilm, although expression levels of *eno* were 1.2-fold higher after 6, 8 and 12 h in biofilm with TC compared to the control (Figure 2A–C).

In the strongly adhering strain, expression levels of *eno* and *ebps* genes at TC concentration of 1/8 MBIC did not significantly differ, and were similar to biofilm without TC (Figure 2A,B). Whereas, the expression of the *fib* gene after 3 and 6 h at the presence of TC (1/8 MBIC) was above half lower than in biofilm without TC. After 8 and 12 h, expression levels of *fib* in biofilm treated with TC accounted for 0.6 and 0.74 of the expression level in biofilm without TC, respectively (Figure 2C). TC at the concentration of 1/2 MBIC reduced the expression levels of the *eno* gene in both biofilms formed by strongly and weakly adhering strain. The highest decrease in expression was observed after 3 and 6 h. In the case of the weak biofilm producer, the values were 3-fold lower in the presence of TC, while in the biofilm of strong producer, this accounted for only 0.1 and 0.2 of expression level in the control. In the next time points, expression levels were significantly higher, but still lower than in the control biofilm (Figure 2A). The expression level of the *ebps* gene in the presence of TC at the concentration of 1/2 MBIC in the weakly adhering strain decreased slowly in the subsequent time points (although did not significantly differ), and after 12 h this expression level was twice lower than in biofilm without TC. In the case of the strongly adhering strain, the highest decrease of *ebps* expression level was observed after 8 h, and it was about 5-fold lower than in untreated biofilm (Figure 2B). The TC at the concentration of 1/2 MBIC also reduced the expression level of the *fib* gene. In the case of weakly adhering strain, the lowest expression was observed after 6 and 8 h, and it was twice lower than in biofilm without TC. In biofilm formed by the strongly adhering strain after 6, 8 and 12 h, the expression level of the *fib* gene was 10-fold lower in the presence of TC (1/2 MBIC) than in biofilm without TC (Figure 2C).

**Figure 3:**
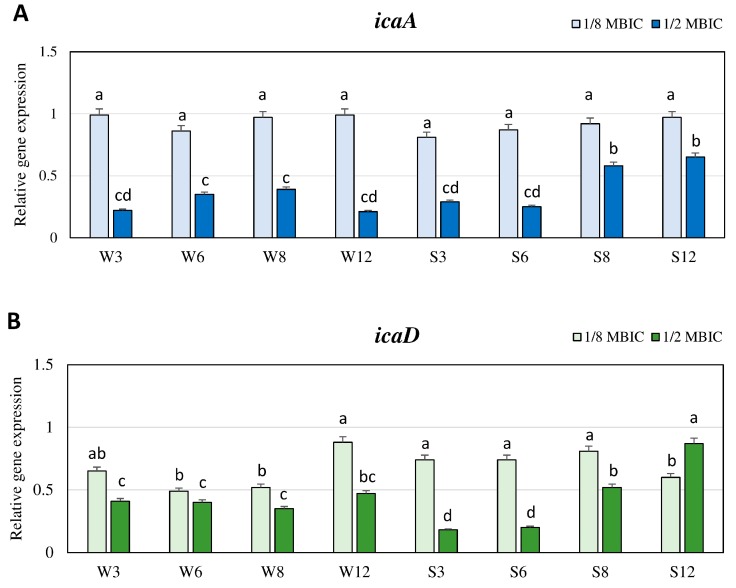
Transcriptional reprogramming of *icaA* (**A**) and *icaD* (**B**) genes in MRSA strains treated with TC under biofilm growth condition. Gene expression data were normalized to the *rpoB* reference gene. Results are shown as *n*-fold changes (mean ± SD) in the target gene expression compared to the control (biofilm untreated with TC). Significant differences in relative gene expression among examined MRSA samples were marked by different letters (a, b, c, d) (Tukey’s test; *p* < 0.05). W: weak producer of biofilm; S: strong producer of biofilm; 3, 6, 8, 12: time of bacterial growth (h); MBIC: minimum biofilm inhibition concentration. Each assay was performed three times and the results were averaged.

The influence of TC on the expression levels of *icaA* and *icaD* genes from the *ica* operon that are involved in the biosynthesis of glucosamine polymer PIA were also evaluated. Expression levels of the *icaA* gene in the presence of TC at the concentration of 1/8 MBIC in the biofilm of both weakly and strongly adhering strains in different time points did not significantly differ, and they were similar as in untreated TC biofilm (Figure 3A).

Whereas, in the case of the *icaD* gene, the expression level in the presence of TC at the concentration of 1/8 MBIC was lower than in the biofilm without TC. The lowest values were observed after 6 and 8 h in the weak biofilm and after 12 h in the strong biofilm (Figure 3B). In the presence of TC at the concentration of 1/2 MBIC, the expression level of *icaA* and *icaD* in the case of the weakly adhering strain was above twice lower than in biofilm without TC, and did not significantly differ in the subsequent time points (Figure 3A,B). In the case of the strongly adhering strain, inhibition of *icaA* and *icaD* expression by TC was highest after 3 and 6 h, and the values were lower (3.5 to 5.5-times) than in the untreated biofilm. After 8 and 12 h, expression levels of these genes significantly increased, but they were still lower than in biofilm without TC (Figure 3A,B).

Importantly, factorial analysis of variance (ANOVA) results indicated that all of the tested variables (strains, treatment, growth period) as well as the vast majority of their interactions, significantly affected the expression levels of five target genes (*icaA*, *icaD*, *eno*, *ebps*, *fib*) and the metabolic activity of MRSA strains (Tables S1 and S2). However, the effect of two interactions (strains × treatment; strains × treatment × growth period) on the expression level of the *ebps* gene in MRSA strains was insignificant (Table S1). Similarly, the effect of interactions of three tested variables (strains × treatment × growth period) on the metabolic activity of the investigated strains appeared to be irrelevant (Table S2).

## 3. Discussion

*S. aureus* is responsible for a wide range of chronic infections, often associated with the formation of biofilm, and for this reason the eradication of *S. aureus*, especially MRSA strains, by classical antimicrobial treatment, is not always successful. Colonization and formation of biofilm by *S. aureus* on medical surfaces such as catheters and other devices is the main problem in the healthcare-associated infections [14]. The lack of therapeutic success in chronic infections is also a consequence of the use of planktonically growing bacteria instead of biofilms in conventional susceptibility tests [15]. Because biofilm infections are difficult to treat, prevention of biofilm formation is an important strategy in biofilm control. The first stage of biofilm formation is attachment of planktonic bacteria to the surfaces of various materials, including host tissues and medical devices, and the accumulation of bacterial cells [16]. Initial attachment to native tissues and biomaterials is mediated by MSCRAMMs that have the ability of binding to one or more host extracellular matrix factors [17,18]. In our earlier study, we showed significantly higher transcript levels of the genes encoding laminin binding protein (*eno*), elastin binding protein (*ebps*) and fibrinogen binding protein (*fib*) in biofilm compared to growth under planktonic conditions. This suggests that these genes are important in the initial phase of biofilm growth, in which bacterial cells interact with host extracellular ligands [5]. Therefore, looking for the alternative factors to antibiotics, that prevent biofilm formation, we investigated the effects of TC on the expression of these genes during biofilm formation by two MRSA strains and on the metabolic activity of bacterial cells in biofilm. TC is a phenolic compound extracted from bark of cinnamon that shows antibacterial activity. Essential for the antibacterial activity of TC is the presence of the acrolein group (α,β-unsaturated carbonyl moiety) [19]. TC as an antimicrobial agent shows low toxicity in a low dose, and is classified as safe for the addition to foods by the US Food and Drug Administration (FDA) [20]. Ribeiro et al. [21], by using a fibroblast cell line, showed that TC did not compromise fibroblast cell viability. Ferro et al. [22], who investigated influence of TC on the survival of *Galleria mellonella* larvae infected with *S. aureus*, showed that this compound improved larval survival due to a reduction of bacterial load in their hemolymph, and obtained no results indicating the toxic effect of TC in doses equivalent to 25–50 mg/kg.

In order to establish a TC concentration effective against bacterial cells forming biofilm, we determined MBIC and investigated the influence of TC sub-inhibitory concentrations on expression levels of the biofilm-associated genes. The obtained results showed that the expression levels of the *eno*, *ebps* and *fib* genes in the presence of TC at the concentration of 1/2 MBIC were lower than in biofilms untreated with TC both in the weakly and strongly adhering strain. The highest decrease in the expression level was observed in the case of the *fib* gene in the strongly adhering strain, in which the amount of *fib* transcript was 10-fold lower compared to biofilm formed by this strain without TC. Our results showed that TC could be an effective factor inhibiting MRSA adhesion to fibrinogen present in blood, a protein mediating platelet adherence and aggregation at the injury site, and being one of main proteins deposited on implanted biomaterials. However, further experiments are required to confirm this effect. Moreover, adhesion of *S. aureus* to fibrinogen is an important factor of wound infection and endocarditis [8].

Transcriptional responses of *eno* and *ebps* genes in the biofilm of MRSA strains treated with TC at the concentration of 1/2 MBIC was also lower than in biofilm without TC, but the number of transcripts was diverse in different time points. The highest decrease in expression of *eno* was observed after 3 and 6 h. In the weak biofilm producer, the expression level was 3-fold lower in the presence of TC and in the biofilm formed by strong producer, and accounted only for 0.1 and 0.2 of expression level in the control, respectively. The highest decrease of *ebps* expression level was observed after 8 h, and it was about 5-fold lower than the expression level of this gene in the biofilm without TC. These results indicate that TC is also an efficient inhibitor of MRSA adhesion to major components of the extracellular matrix such as elastin and laminin, which prevents dissemination of staphylococcal cells and the initiation of host tissue colonization. TC is widely known for its antibacterial properties [23,24,25,26,27,28,29,30,31] but to our knowledge, the influence of this compound on the expression of genes associated with biofilm formation by *S. aureus* was not yet investigated.

The action of TC results in damage of the cell wall and membrane of treated bacteria, and consequently to the loss of inner cell material, cell lysis and leakage of cellular contents [32,33]. Mousavi et al. [34] showed also that TC acts as an oxidative stress agent. Unlike antibiotics, TC changes bacterial metabolism through interaction with several different biochemical families such as proteins, nucleic acids, lipids and carbohydrates [34], which also indicates that the emergence of an effective mechanism of resistance to this compound is very difficult to achieve. Our results showed that TC is effective as a factor inhibiting the expression of genes encoding adhesive proteins that are necessary to the binding of staphylococcal cells to surface, and they expand knowledge about the mechanisms of TC action. In our study, we also investigated influence of TC on the expression of *icaA* and *icaD* genes responsible for intercellular adherence and accumulation of bacterial cells in biofilm. Similarly, as in case of adhesin genes, the TC at concentration of 1/2 MBIC inhibited the expression of *icaA* and *icaD* genes. In the weakly adhering strain the expression level was above twice lower than in biofilm without TC, whereas in the strongly adhering strain, a similar decrease of expression was obtained only after 3 and 6 h. Significant increase in the expression level of *icaA* and *icaD* after 8 and after 12 h in the case of the strongly adhering strain was probably related to rapid cell division that resulted in the thick cell and slime layer, hardly penetrated by TC. Higher expression of *eno* and *ebps* genes and metabolic activity were also observed after 12 h in this strain, although it was lower than in untreated biofilm.

Our results are similar to those obtained by Ferro et al. [22], who also found that 1/2 MIC of TC reduced *S. aureus* metabolic activity in biofilm. In our study, reduction of metabolic activity in the presence of TC at 1/2 MBIC reached over 50%. Studies by other authors showed the synergistic effect of TC with antibiotics, which allowed us to obtain antimicrobial activity at lower concentrations of these substances. The combinations of TC and antibiotics can be used as an alternative therapeutic application, which can decrease the minimum effective dose of the drugs, thus reducing their possible adverse effects and the costs of treatment. Reduction of antibiotic concentration may help to prevent the spread of antibiotic resistance [26,29,35,36]. In the case of the weakly adhering strain, reduction of biofilm metabolic activity at the presence of 1/2 TC MBIC after 3, 6 and 8 h did not significantly differ from the strongly adhering strain. After 12 h, metabolic activity of the weakly adhering strain at the presence of TC was significantly lower than in the strong biofilm producer. A sublethal concentration of TC did not affect the integrity of the membrane, but it did inhibit the growth of cells, which indicates that TC gained access to the cytoplasm [26]. Antimicrobial action of TC is also related to the inhibition of cell division through inhibiting of (GTP)-dependent FtsZ polymerization (filamentation temperature sensitive protein Z) [37], thus the effect of lower metabolic activity is more evident in the slowly growing strain. In this study, TC at the concentration of 1/8 MBIC also reduced metabolic activity, although the degree of reduction was higher in the case of the weakly adhering strain. Whereas, in the case of this strain, slightly higher expression of the *eno* gene in the presence of TC at 1/8 MBIC compared to biofilm without TC was observed. Many authors [9,38,39,40,41], reported that biofilm formation can be induced by conditions that are potentially toxic to bacterial cells, such as anaerobic conditions, ethanol and the presence of sub-MICs of some antibiotics. Schilcher et al. [41] suggests that this effect is strain-specific and related to the induction of stress pathways in *S. aureus* that leads to expression of genes associated with biofilm formation. Whereas, the results of our study also showed that the presence of TC at the concentration of the 1/8 MBIC expression level of the *icaA* gene in both the weakly and strongly adhering strain, was similar to expression in untreated TC biofilm. Different results were obtained by Nuryastuti et al. [42], who reported that sub-MIC concentrations of cinnamon oil strongly increased the expression of the *icaA* gene in clinical *S. epidermidis* strains.

In our study, we obtained different values of MIC, MBC, MBIC and MBEC for two tested strains. Variability between clinical isolates was also observed in other studies—MIC of TC for *S. epidermidis* isolates were 400–500 µg/mL [29]. Other research concerning antimicrobial activity of TC showed that MIC values for *S. aureus* isolates were higher than in our study (250 µg/mL) [22]. These results support our findings, showing diversity in the sensitivity on TC within the species.

Eradication of mature biofilm with both antibiotics and other substances is very difficult as evidenced by the high concentration of TC required for biofilm eradication (MBEC). However, in our study, we showed that low TC concentrations (1/2 MBIC) prevent biofilm formation by reducing metabolism and the levels expression of several genes associated with biofilm formation. Our results showed that TC can be used as an antibacterial agent for external use or to protect biomaterials, e.g., as a component of coatings on catheters that prevent biofilm formation.

## 4. Materials and Methods

### 4.1. Bacterial Strains

Two methicillin-resistant *Staphylococcus aureus* (MRSA) strains isolated from a wound and the anus of hospitalized patients in 2017 were used in this research. The strains were obtained from a hospital in Siedlce (Poland). Methods of identification of genes: *mecA* responsible for resistance against β-lactam antibiotics, encoding laminin binding protein (*eno*), elastin binding protein (*ebps*) and fibrinogen binding protein (*fib*) encoding microbial surface components recognizing adhesive matrix molecules (MSCRAMMs) and genes from the *ica* operon (*icaA* and *icaD*) were described earlier [5]. The strain 27887 isolated from the wound formed strong biofilm on polystyrene, while the strain from the anus weakly adhered to polystyrene [5].

### 4.2. Determination of Minimum Inhibitory Concentrations (MIC) and Minimum Bactericidal Concentrations (MBC) of TC

Antimicrobial effects of *trans*-cinnamaldehyde (TC) (Sigma-Aldrich, Steinheim, Germany, lot no. MKBW8907V) were evaluated using a serial twofold dilution method. TC was initially diluted in dimethyl sulfoxide (DMSO) (Sigma-Aldrich) (1:1 *v*/*v*) and then in Mueller-Hinton Broth (MHB; BBL, Becton Dickinson, Sparks, Md., Franklin Lakes, NJ, USA). Serial twofold dilutions for TC were prepared to obtain concentrations ranging from 50 to 0.002 mg/mL. Determination of MIC and MBC of TC was carried out according to method described by Kot et al. [43].

### 4.3. Determination of Minimum Biofilm Inhibition Concentrations (MBIC) and Minimum Biofilm Eradication Concentrations (MBEC) of TC

Antimicrobial effects of TC against biofilm was evaluated using a serial twofold dilution method. TC was initially diluted in DMSO (1:1 *v*/*v*), and then in Tryptic-Soy Broth (TSB; BBL, Becton Dickinson, Sparks, Md.) with 0.5% glucose. Serial twofold dilutions for TC were prepared to obtain concentrations ranging from 50 to 0.012 mg/mL. Aliquots of 100 µL of tested TC concentrations were transferred in three replicates to wells of tissue culture polystyrene 96-well plate (Nunclon, Roskilde, Denmark). *S. aureus* strains were grown on Tryptic-Soy Agar (TSA; BBL, Becton Dickinson, Sparks, Md.) with 0.5% glucose at 37 °C for 18 h. Bacterial cells of each strain were suspended in sterile phosphate-buffered saline (PBS, pH 7.4) to get optical density OD_565_ = 3.2 (densitometer DEN-1, Biosan, Riga, Latvia). The obtained suspensions of bacterial cells were diluted 1:10 (*v*/*v*) with TSB with 0.5% glucose in order to prepare a cell suspension containing about 10^8^ CFU/mL. Bacterial suspensions were transferred (100 µL) to the wells of microplates containing TC solutions. Control of bacterial growth was performed in the wells with cell suspensions without TC, as well as control of the sterility of the media (wells without addition of cell suspension). Control of bacterial growth was also performed in the wells with bacterial cell suspensions with DMSO and no inhibitory effects were found with DMSO at 5% (*v*/*v*). Inoculated microplates were incubated without agitation at 37 °C for 24 h. Determination of the minimum biofilm inhibition concentration (MBIC) values of TC was carried out by the resazurin microtiter-plate assay that allowed us to determine metabolic activity of bacterial cells. To evaluate the susceptibility of bacteria in biofilm to antibiotics and other antimicrobial compounds, it is essential to determine the amount of viable bacteria in the biofilm [44].

Metabolic assay is excellent for the quantification of bacterial viability in biofilm, and the amount of metabolite produced by a biofilm depends on both the metabolic activity of the individual bacteria and the number of live bacteria in the biofilm. The resazurin assay (the Alamar Blue assay) has been described as a reliable and reproducible method for evaluating biofilm susceptibility [21,45,46]. The resazurin assay is based on the reduction of resazurin, a blue dye that can be reduced by metabolically active cells to pink resorufin, which is fluorescent. Measurement of fluorescein content in a biofilm is useful to quantify the viability of the biofilm cells after antimicrobial treatment [47,48]. For this purpose, after the incubation period, the medium was removed, and non-adherent bacterial cells were discarded by washing the biofilms twice with 250 µL of sterile PBS. Subsequently, TSB with 0.5% glucose (190 µL) and 10 µL of resazurin (Sigma-Aldrich) aquatic solution (0.01%) were added to each well with biofilm. Then the microplates were incubated for 2 h in darkness at 37 °C. The change of color from blue to pink indicated a reduction of resazurin by live bacterial cells. The lowest concentration of TC at which resazurin color change was not observed, which confirmed inhibition of the metabolic activity of bacterial cells by TC, was taken as the MBIC value. Each test was repeated three times. For the purpose of determining the MBEC, TSB with 0.5% glucose, containing tested TC concentrations, was inoculated in triplicate, in a volume of 200 µL in the wells with established biofilm and incubated for 24 h at 37 °C. Biofilm formation assay was performed as described by Kot et al. [25]. Subsequently, the medium was removed, and the biofilms were washed twice with 250 µL of sterile PBS. Then, TSB with 0.5% glucose (190 µL) and 10 µL of resazurin were added to each well with biofilm earlier treated with TC, and the microplates were incubated for 2 h in darkness at 37 °C. The lowest concentration of TC at which no resazurin color change was observed, was taken as the MBEC value. For each strain, three control wells with biofilms containing TSB with 0.5% glucose without TC were also included.

### 4.4. TC Inhibiting Biofilm Formation Assay in Different Time Intervals

To evaluate the effectiveness of TC against biofilm formation, tissue culture polystyrene 96-well plates were used. Suspensions of bacterial cells in PBS (pH 7.4) were diluted in order to prepare suspensions containing about 10^8^ CFU/mL in TSB with 0.5% glucose and TC at final concentrations of 1/8 MBIC and 1/2 MBIC. After that, bacterial cell suspensions (200 µL) were transferred in four replicates to wells of polystyrene plate. The plates were incubated at 37 °C for 3, 6, 8 and 12 h without agitation. Control of bacterial growth was performed in the wells with bacterial cell suspensions without TC, as well as control of sterility of the media (wells without addition of cell suspension). After the appropriate incubation period, metabolic activity of bacterial cells in biofilm was determined by the resazurin microtiter-plate assay, as described above. After incubation of plates with resazurin, the absorbance was measured at 595 nm in microplate reader (Apollo LB913, Berthold Technologies, Bad Wildbad, Germany). Each assay was performed three times and the results were averaged. The influence of TC on the metabolic activity of biofilms was evaluated by comparison to the metabolic activity of biofilms that were not exposed to TC. The results are presented as percentages of biofilm metabolic activity reduction, according to Borges et al. [49].

### 4.5. Growth Conditions of MRSA Strains

Two MRSA strains were grown in TSB with 0.5% glucose at 37 °C for 18 h. Subsequently, the cultures were inoculated on TSA with 0.5% glucose and incubated at 37 °C for 18 h. After that, bacterial cells of each strain were suspended in the PBS (pH 7.4) to get the optical density OD_565_ = 3.2. The obtained suspensions were diluted with TSB with 0.5% glucose and TC at final concentrations of 1/8 MBIC and 1/2 MBIC in order to prepare cell suspensions containing about 10^8^ CFU/mL. For biofilm growth conditions, bacterial cell suspensions with TC (1 mL) were transferred in eight replicates to wells of a tissue culture polystyrene 24-well plate (Nunclon, Roskilde, Denmark) and incubated at 37 °C. Control of bacterial growth was performed in the wells with bacterial cell suspensions in TSB with 0.5% glucose.

### 4.6. Preparation of the Lysate from Bacterial Cells, RNA Extraction and cDNA Synthesis

Biofilm-grown bacterial cells in the presence of and without TC were harvested at four different times (3, 6, 8 and 12 h). The biofilm cells were rinsed with sterile water, scraped with a pipette and suspended in an appropriate volume of RNAprotect Bacteria Reagent (QIAGEN, Hilden, Germany) in order to prepare cell suspensions containing about 5 × 10^8^ CFU/mL.

Preparation of the lysate from bacterial cells, RNA extraction and cDNA synthesis were performed according to method described earlier by Kot et al. [5].

### 4.7. Gene Quantification

Quantification of *icaA*, *icaD*, *eno*, *ebps* and *fib* genes of MRSA strains was performed using a quantitative real-time polymerase chain reaction (qRT-PCR) technique according to the protocol presented earlier by Kot et al. [5]. The list of primers and TaqMan fluorescent probes that were used in the study is presented in Table 2. The *RpoB* gene, encoding the RNA polymerase subunit, was used as the internal reference.

### 4.8. Statistical Analysis

All data were expressed as the mean (± standard deviation (SD)). The obtained results were assessed with the use of STATISTICA 12 software (StatSoft, Poland). Three-factorial analysis of variance (ANOVA) with consecutive Tukey’s test were applied to evaluate the significance of the effects of tested variables (strain, treatment, growth period) and their interactions on the relative expression levels of five target genes (*icaA*, *icaD*, *eno*, *ebps*, *fib*) of MRSA strains. Three-factorial analysis of variance (ANOVA) with consecutive Tukey’s test were also used to evaluate the significance of effects of tested variables (strain, treatment, growth period) and their interactions on the metabolic activity of MRSA strains.

## 5. Conclusions

The results obtained in this study showed the new mechanism of TC action involved in the preventing of biofilm formation. Metabolic activity of *S. aureus* cells in biofilm and the expression levels of genes that are involved in the synthesis of binding factors and PIA were significantly reduced in the presence of TC at 1/2 MBIC already in the first hours of biofilm formation.

The action of TC at sub-inhibitory concentrations included a significant decrease in the expression levels of *eno*, *ebps* and *fib* genes encoding binding proteins that may prevent colonization of host tissues or foreign materials. TC also prevented biofilm structure formation by inhibiting of the expression of the genes encoding glucosamine polymer PIA involved in development of multiple layers of sessile bacterial cells protected by a slime substance. This mechanism of TC action may reduce resistance of bacterial cells to antibiotics and host immune response. Our study demonstrated strong activity of TC against biofilm formed by clinical MRSA strains, and therefore this compound has a therapeutic potential as an antibacterial agent for external use or to protect biomaterials, e.g., as a component of coatings on catheters to prevent biofilm formation.

## Figures and Tables

**Table 1:** Susceptibility to *trans*-cinnamaldehyde (TC) of methicillin-resistant *Staphylococcus aureus* (MRSA) strains in planktonic culture and biofilm conditions.

Strain	Source	Biofilm	*trans*-Cinnamaldehyde (mg/mL)			
			Planktonic Cells		Biofilm	
			MIC	MBC	MBIC	MBEC
27,887	Wound	Strong	0.24	0.48	0.48	0.96
1037	Anus	Weak	0.06	0.06	0.12	1.92

MIC—minimum inhibitory concentration, MBC—minimum bactericidal concentration, MBIC—minimum biofilm inhibition concentration, MBEC—minimum biofilm eradication concentration.

**Table 2:** Sequences of primers designed for quantitative real-time polymerase chain reaction (qRT-PCR) analyses.

Genes	Accession No. (GenBank)	Sequences of Primers and Probes
*icaA*	SAB2541 (K11936)	F: CAATACTATTTCGGGTGTCTTCACTCT R: CAAGAAACTGCAATATCTTCGGTAATCAT P: 5′-FAM-CCCAGTAGCCAACATC-NFQ-3′
*icaD*	SAB2542 (K21461)	F: TCAAGCCCAGACAGAGGGAATA R: ACACGATATAGCGATAAGTGCTGTTT P: 5′-FAM-CCCAACGCTAAAATC-NFQ-3′
*eno*	AF065394.1	F: AAACTGCCGTAGGTGACGAA R: TGTTTCAACAGCATCTTCAGTACCTT P: 5′-FAM- TTCGCTCCTAAATTTG-NFQ-3′
*ebps*	SAB1343c	F: ACATTCAAATGACGCTCAAAACAAAAGT R: CTTATCTTGAGACGCTTTATCCTCAGT P: 5′-FAM- CAAGGCGAATAACTCG-NFQ-3′
*fib*	SAB1021 (K14200)	F: GAATATGGTGCACGTCCACAATT R: AAGATTTTGAGCTTGAATCAATTTTTGTTCTTTTT P: 5′-FAM-TCGCTGCTGGTTTATT-NFQ-3′
*rpoB*	KY086792.1	F: CAGCTGACGAAGAAGATAGCTATGT R: ACTTCATCATCCATGAAACGACCAT P: 5′-TAGCACAAGCAAACTC-NFQ-3′

## Supplementary Materials

The following are available online at https://www.mdpi.com/1422-0067/21/1/102/s1.
