# Supplementary material for: Effect of trans-Cinnamaldehyde on Methicillin-Resistant Staphylococcus aureus Biofilm Formation: Metabolic Activity Assessment and Analysis of the Biofilm-Associated Genes Expression
Source: Int J Mol Sci. 2019 Dec 22;21(1):102. doi: 10.3390/ijms21010102 (PMC6981724; doi:10.3390/ijms21010102)
Supplement: Supplementary file 1 [file ijms-21-00102-s001.pdf]

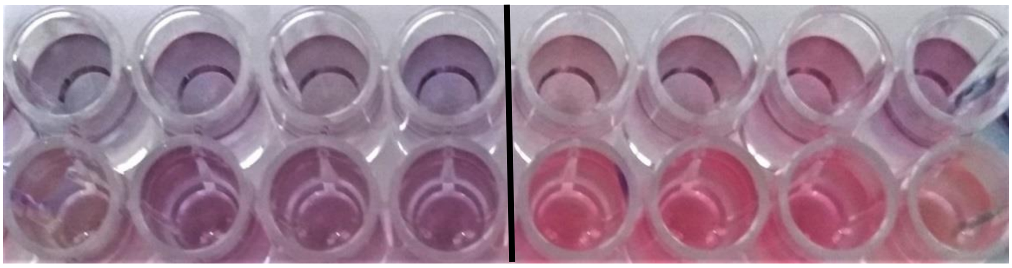

Figure S1. Effect of TC at concentration ½ MBIC on MRSA biofilm metabolic activity after 3 h of treatment, evaluated by the resazurin microtiter-plate assay. The quantitative analysis of biofilm metabolic activity was determined by measurement (at 595 nm) of the optical density of biofilms containing 0.01% resazurin.

Table S1. Three-factorial ANOVA analysis of the studied variables (strains, treatment, growth period) and interactions on relative expression of the examined genes (*icaA*, *icaD*, *eno*, *ebps*, *fib*) of MRSA strains.

Variables	*icaA*	*icaD*	*eno*	*ebps*	*fib*
Strains (S)	$F_{1, 48}$ = 524 (***)	$F_{1, 48}$ = 112 (**)	$F_{1, 48}$ = 2,108 (***)	$F_{1, 48}$ = 75 (*)	$F_{1, 48}$ = 803 (***)
Treatment (T)	$F_{2, 48}$ = 9,157 (***)	$F_{2, 48}$ = 1,831 (***)	$F_{2, 48}$ = 7,120 (***)	$F_{2, 48}$ = 134 (*)	$F_{2, 48}$ = 445 (***)
Growth period (G)	$F_{3, 48}$ = 163 (**)	$F_{3, 48}$ = 355 (***)	$F_{3, 48}$ = 129 (**)	$F_{3, 48}$ = 208 (**)	$F_{3, 48}$ = 177 (**)
S × T	$F_{2, 48}$ = 315 (***)	$F_{2, 48}$ = 670 (***)	$F_{2, 48}$ = 92 (*)	$F_{2, 48}$ = 2 (ns)	$F_{2, 48}$ = 28 (*)
T × G	$F_{6, 48}$ = 120 (**)	$F_{6, 48}$ = 178 (**)	$F_{6, 48}$ = 14 (*)	$F_{6, 48}$ = 61 (*)	$F_{6, 48}$ = 45 (*)
S × G	$F_{3, 48}$ = 38 (*)	$F_{3, 48}$ = 99 (*)	$F_{3, 48}$ = 25 (*)	$F_{3, 48}$ = 19 (*)	$F_{3, 48}$ = 622 (***)
S × T × G	$F_{6, 48}$ = 104 (*)	$F_{6, 48}$ = 81 (*)	$F_{6, 48}$ = 6 (*)	$F_{6, 48}$ = 1 (ns)	$F_{6, 48}$ = 18 (*)

* P<0.05; ** P<0.01; *** P<0.001; ns – non-significant. Variability source: i) strains – weak and strong biofilm formation; ii) treatment: control, ⅛ and ½ MBIC; iii) growth period – 3, 6, 8 and 12 h.

Table S2. Three-factorial ANOVA analysis of the studied variables (strains, treatment, growth period) and interactions on the metabolic activity of MRSA strains.

**Variables**	**Metabolic activity**
Strains (S)	$F_{1, 48}$ = 125 (**)
Treatment (T)	$F_{2, 48}$ = 247 (***)
Growth period (G)	$F_{3, 48}$ = 98 (**)
S × T	$F_{2, 48}$ = 6 (*)
T × G	$F_{6, 48}$ = 11 (*)
S × G	$F_{3, 48}$ = 15 (*)
S × T × G	$F_{6, 48}$ = 2 (ns)

* *P*<0.05; ** *P*<0.01; *** *P*<0.001; ns – non-significant. Variability source: i) strains – weak and strong biofilm formation; ii) treatment – control, ⅛ and ½ MBIC; iii) growth period – 3, 6, 8 and 12 h.
